# Capability, opportunity, and motivation to enact hygienic practices in the early stages of the COVID‐19 outbreak in the United Kingdom

**DOI:** 10.1111/bjhp.12426

**Published:** 2020-05-16

**Authors:** Jilly Gibson Miller, Todd K. Hartman, Liat Levita, Anton P. Martinez, Liam Mason, Orla McBride, Ryan McKay, Jamie Murphy, Mark Shevlin, Thomas V. A. Stocks, Kate M. Bennett, Richard P. Bentall

**Affiliations:** ^1^ University of Sheffield UK; ^2^ University College London UK; ^3^ Ulster University UK; ^4^ Royal Holloway University of London UK; ^5^ University of Liverpool UK

**Keywords:** COVID‐19, COM‐B, behaviour change, hygienic practices, pandemic

## Abstract

**Objectives:**

The COVID‐19 pandemic is one of the greatest global health threats facing humanity in recent memory. This study aimed to explore influences on hygienic practices, a set of key transmission behaviours, in relation to the Capability, Opportunity, Motivation‐Behaviour (COM‐B) model of behaviour change (Michie et al., 2011).

**Design:**

Data from the first wave of a longitudinal survey study were used, launched in the early stages of the UK COVID‐19 pandemic.

**Methods:**

Participants were 2025 adults aged 18 and older, representative of the UK population, recruited by a survey company from a panel of research participants. Participants self‐reported motivation, capability, and opportunity to enact hygienic practices during the COVID‐19 outbreak.

**Results:**

Using regression models, we found that all three COM‐B components significantly predicted good hygienic practices, with motivation having the greatest influence on behaviour. Breaking this down further, the subscales psychological capability, social opportunity, and reflective motivation positively influenced behaviour. Reflective motivation was largely driving behaviour, with those highest in reflective motivation scoring 51% more on the measure of hygienic practices compared with those with the lowest scores.

**Conclusions:**

Our findings have clear implications for the design of behaviour change interventions to promote hygienic practices. Interventions should focus on increasing and maintaining motivation to act and include elements that promote and maintain social support and knowledge of COVID‐19 transmission. Groups in particular need of targeting for interventions to increase hygienic practices are males and those living in cities and suburbs.

**Statement of Contribution**

***What is already known on this subject?***

- The citizens of the world have been asked to make significant and urgent changes in behaviour to stem the spread of COVID‐19.
- The COM‐B model of behaviour change provides a useful theoretical framework for understanding the influences on behaviours in depth.
- It is important to explore the determinants of behaviour in the unprecedented current socio‐economic climate, to inform behaviour change interventions.

***What does this study add?***

- This study provides insight into the factors influencing UK citizens’ hygienic practices during the early stages of the COVID‐19 pandemic.
- We found that reflective processes were largely driving hygienic practices – these involve making plans to enact the behaviour and supporting the belief that the behaviour is a good thing to do.
- Behaviour change interventions to improve and maintain hygienic practices throughout the lockdown and beyond should contain behaviour change techniques that focus on self‐regulatory processes involving planning and goal setting.

The COVID‐19 pandemic began in China in late 2019 and is perhaps one of the biggest health threats the world has faced this century. As of mid‐April 2020, there have been over 2 million confirmed cases of COVID‐19 and nearly 150,000 deaths globally (WHO, 2020). As we write, death rates from the coronavirus in the United Kingdom are increasing daily and are currently over 16,000 (PHE, 2020). From 23 March 2020, strict social distancing measures were put in place for UK citizens in order to slow down the spread of the virus, including laws to allow the police to enforce the measures (Cabinet Office, 2020). These measures have been accompanied by increases in unemployment, catastrophic financial loss for many citizens, and predictions of severe economic impacts (The Office for Budget Responsibility: OBR, 2020). Further, citizens face great uncertainty along with immediate‐ and long‐term costs to mental health (Brooks *et al.*, 2020; Shevlin *et al.*, 2020). The National Health Service (NHS) is under great pressure in providing care for hospitalized patients, for which there are national shortages of personal and protective equipment, and as we write, the Prime Minister, Boris Johnson, is currently recovering from COVID‐19 after receiving emergency care in hospital. The country is, in short, in the midst of the worst public health crisis in recent history.

Human behaviour will play a decisive role in the shape and spread of the COVID‐19 infection, and the speed of the spread across the world. Behavioural science has a central role to play in understanding the mechanisms that drive people to enact behaviours that will essentially shape the progression of the outbreak (Michie, Rubin & Amlot, 2020). A key set of behaviours recommended during the outbreak of COVID‐19 to help prevent infection and slow the spread of disease are maintaining hygienic practices (e.g., handwashing frequently, cleansing surfaces, and using tissues). These measures were recommended to UK citizens from the start of the outbreak, around February 2020, and signified large‐scale and urgent changes in behaviour that may be psychologically burdensome to successfully enact for individuals (Van Bavel *et al.*, 2020).

The British Psychological Society (BPS) Behavioural Science and Disease Prevention Taskforce (BSDPT:, 2020) recommends the exploration of behavioural influences on enacting preventive behaviour in relation to the Capability, Opportunity, Motivation‐Behaviour (COM‐B) model of behaviour change (Michie *et al.*, 2011). This model proposes that a person must have sufficient psychological and physical capability (strength, knowledge, skills, etc.), physical and social opportunity (time, social cues, etc.), and reflective and automatic motivation (intentions, planning, emotion regulation, etc.) in order for behaviour to occur. The COM‐B model is at the centre of the Behaviour Change Wheel (BCW), which is a tool kit for designing behaviour change interventions (Michie *et al.*, 2014). Thus, applying the COM‐B model to key COVID‐19 transmission‐related behaviours will enable us to understand these within a theoretical framework. From such a ‘behavioural diagnosis’, we can identify the components that are most likely to influence the behaviour (Michie *et al*, 2011) and, thus, identify appropriate targets for behaviour change interventions (BCIs), which can be designed to improve adherence to preventive behaviours during the period of social isolation.

The present study reports findings from a panel survey, launched 52 days after the first confirmed case of COVID‐19 in the United Kingdom, developed (in part) to understand the underlying drivers of health‐protective behaviours during the COVID‐19 outbreak. The survey explored UK citizens’ motivation, capability, and opportunity to engage in hygienic practices, and took corresponding measures of behaviour.

## Methods

### Participants and procedure

Participants were 2025 adults aged 18 and older, recruited by a survey company (Qualtrics) from a panel of research participants. Quota sampling methods ensured that the sample was representative of the UK population for this age group in terms of age, sex, and gross household income. Panel members were provided with information about this study and asked to participate via email by Qualtrics. Panel members were not obliged to take part but were reimbursed for their time by Qualtrics. Those who chose to participate followed a link to a secure website and completed the survey online, after providing informed consent. Ethical approval for this research was provided by a UK University (Reference Number: 033759).

### Measures

The measures reported here were part of the first cross‐sectional wave of a larger longitudinal survey conducted by the COVID‐19 Psychological Research Consortium (C19PRC) to explore the psychological and social consequences of the COVID‐19 epidemic on the UK population (for full methodology, see McBride *et al.*, 2020). In brief, the survey took measures of socio‐demographic characteristics; health characteristics and behaviour; knowledge, attitudes, and beliefs in relation to COVID‐19; mental health indicators; social attitudes; and psychological variables.

Items relevant to this paper were based on the COM‐B model of behaviour change (Michie *et al.*, 2011) in relation to maintaining hygienic practices, a set of key COVID‐19 transmission‐related behaviours. Items were adapted from a preliminary version of the COM‐B self‐evaluation questionnaire and other guidelines (COM‐B‐Qv1; Michie *et al.*, 2014; West *et al.*, 2019), and respondents indicated the extent to which seventeen statements were true for them during the COVID‐19 pandemic on a 5‐point scale (labelled: strongly agree, agree, neither agree nor disagree, disagree, and strongly disagree). Three items measured psychological capability: For example, ‘I knew about why it was important and had a clear idea about how the virus was transmitted’ (α = .79). Two items measured physical opportunity: For example, ‘It was easy for me to do it’ (α = .85); and four items measured social opportunity: For example, ‘I had support from others’ (α = .71). Five items measured reflective motivation: For example, ‘I intended to do it’ (α = .81); and three items measured automatic motivation: For example, ‘I felt like I could control my emotional reactions so I could do it’ (α = .59). Behavioural measurements were five self‐reported practices in relation to maintaining hygienic practices: touching eyes or mouth, washing hands with soap and water more often, using hand sanitizing gel if soap and water were not available, using disinfectants to wash surfaces in your home more frequently, and covering nose and mouth with a tissue or sleeve when coughing or sneezing. Response scales were ‘No’, ‘Occasionally’, and ‘Whenever possible’. A full list of items is available in Table S1, Appendix S1. For ease of interpretation and comparability across predictors in the analyses, all variables were rescaled to range from 0 to 1.

## Results

Table S2 (Appendix S2) summarizes statistics for all of the variables used in these analyses. To explore the influence of the COM‐B model components on the enactment of hygienic practices, we regressed hygienic practices on the COM‐B components and control variables (age, gender, ethnicity, level of education, income, religious beliefs, place and type of residence, and personal risk of COVID‐19) using ordinary least squares (OLS). The model was statistically significant (*F* (19, 1931) = 25.82, *p* < 0.001); results of this regression are presented in Figure S1 and Table S3 (Appendix S3). All three COM‐B components significantly predicted hygienic practices in the expected (positive) direction: Capability (*b* = .13; 95% CI = 0.05, 0.21; *p* < .01); Opportunity (*b* = 0.22; 95% CI = 0.12, 0.32; *p* < .001); and Motivation (*b* = .43; 95% CI = 0.33, 0.53; *p* < .001). Motivation had the largest influence on behaviour, with those highest in motivation scoring 43% more on the measure of hygienic practices compared with those with the lowest scores, after accounting for control variables. This analysis also illustrated that those who were non‐White, had lower or moderate education levels, and had higher incomes were more likely to engage in hygienic practices, though the effect sizes for these influences on behaviour were smaller than those of the COM‐B constructs. In addition, males, those living in cities and suburbs, and respondents who were non‐religious and non‐Christian were less likely to engage in hygienic practices. Surprisingly, personal risk of COVID‐19 (i.e., those who reported pre‐existing medical conditions or were pregnant) did not predict hygienic practices.

**Figure 1:**
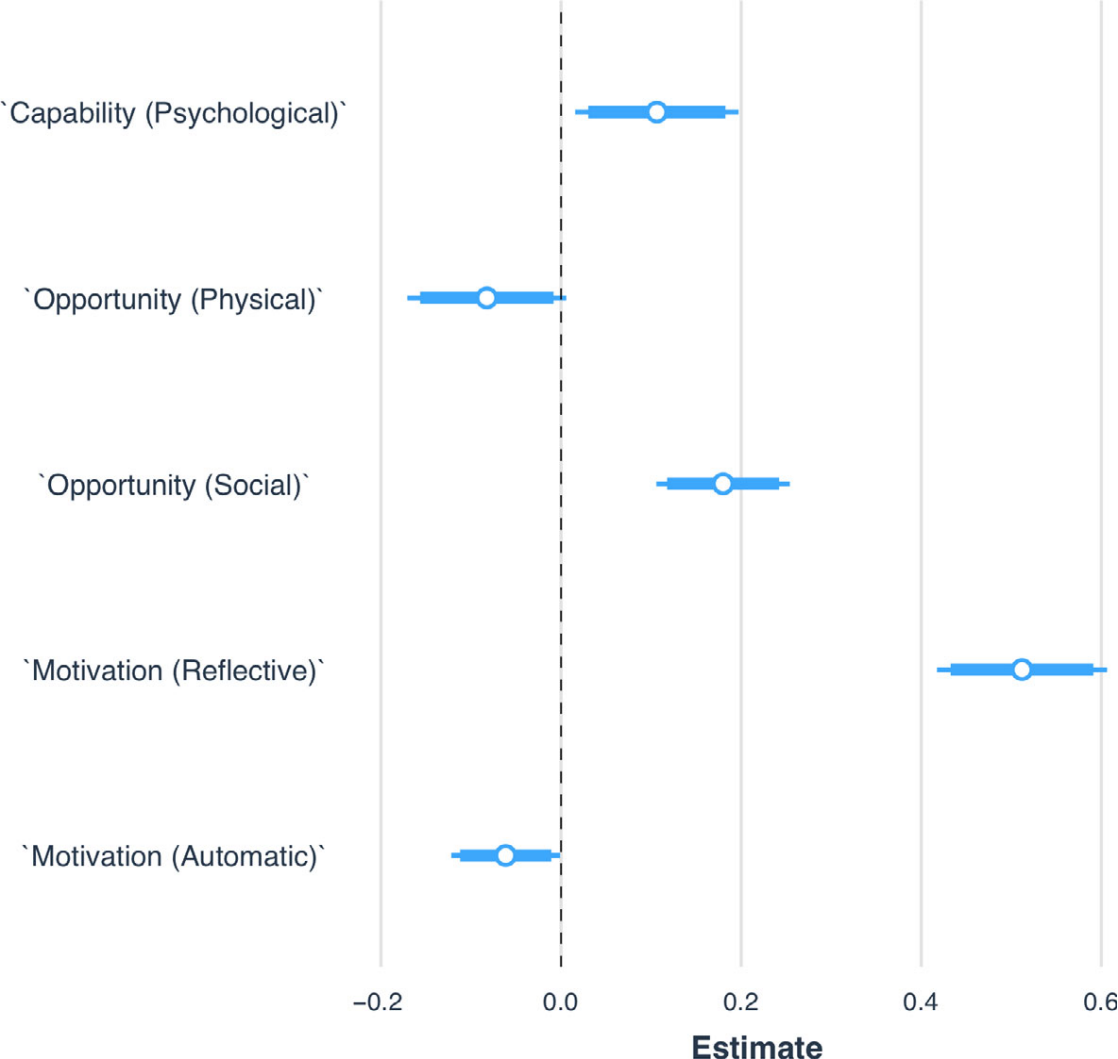
Effects of COM‐B Subscales on Hygienic Practices. *Notes:* Plot points are unstandardized regression coefficients from an OLS model; 95% and 90% confidence intervals indicated by the narrow and thick error bars, respectively. *Notes.* Plot points to the right of the vertical line indicate an increase in hygienic practices; those to the left a decrease. To aid in interpretation, all predictors have been rescaled from 0 to 1

To explore COM‐B influences on behaviour further, we regressed hygienic practices on COM‐B subscales (i.e., psychological capability, physical and social opportunity, and reflective and automatic motivation) using OLS. Results are presented in Figure 1 (below) and Table S4 (Appendix S4). Each of the COM‐B subscales significantly predicted hygienic practices, except for physical opportunity (*b *= −.08; 95% CI = −0.17, 0.01; *p *< .10). Notably, automatic motivation and physical opportunity negatively influenced behaviour, indicating that these components were associated with a decrease in hygienic practices, whilst psychological capability, social opportunity, and reflective motivation increased these behaviours. Moreover, reflective motivation had the largest effect size relative to the other COM‐B subscales, accounting for an increase of more than half of the entire scale of the outcome behaviour (*b* = .51; 95% CI = 0.42, 0.61; *p* < .001).

**Figure 2:**
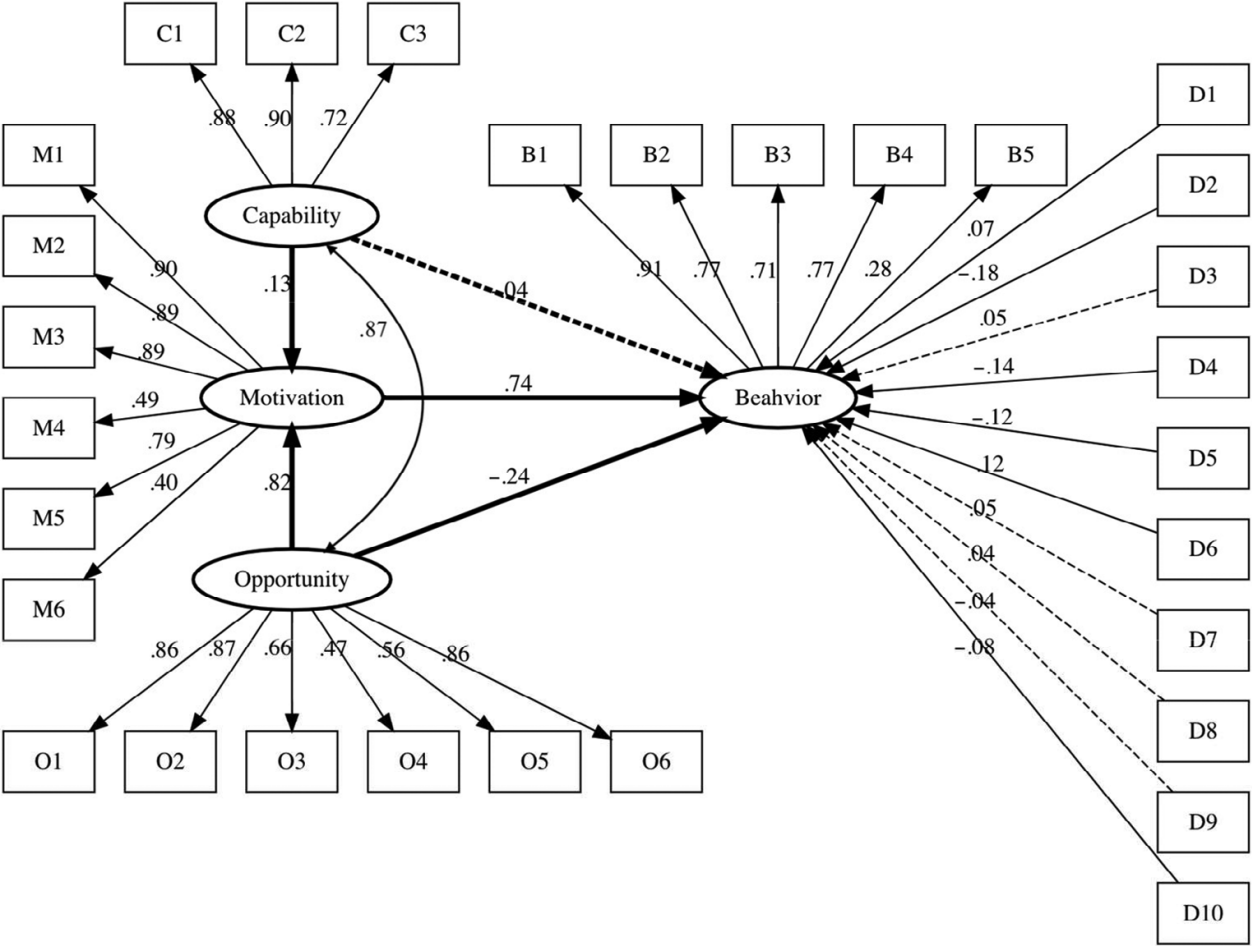
Structural equation model of hygienic practices, COM‐B latent factors, and socio‐demographic characteristics. *Notes: N* = 2,025. Diagonally weighted least squares (DWLS) estimator with robust standard errors using in the ‘lavaan’ package in R. Standardized estimates presented. All paths are statistically significant (*p < *0.05) except for those indicated with a dashed line. RMSEA = .069 [.067, .071], CFI = .95, TLI = .97. D1 = age; D2 = male; D3 = non‐White; D4 = non‐Christian; D5 = non‐religious; D6 = income; D7 = low education; D8 = moderate education; D9 = city; D10 = suburb

To capture the more complex nature of the COM‐B model, as well as account for measurement error, we estimated a structural equation model (SEM) using diagonally weighted least squares (DWLS) and robust standard errors with the ‘lavaan’ package in R. The use of DWLS estimation was an efficient way of handling the categorical variables. The results of this model are presented in Figure 2 below and Appendix S5. To improve model fit, we retained only those control variables that were statistically significant predictors from the regression models (see Table S3). We also dropped two items from the COM‐B motivation scale that were reverse‐coded and had poor overall fit with the latent construct (M6 and M7 in Table S1).

Fit statistics indicate that the SEM fits the data well: RMSEA = .069 [.067, .071], CFI = .95, TLI = .97, *Χ* ^2^(354) = 3740.54, *p* < .001. All estimates were standardized for comparability across scales. Motivation had a large, positive, and statistically significant direct effect on Behaviour (*b* = .74, *p *< .001). However, Opportunity (*b *= −.24, *p *< .05) and Capability (*b *= −.04, *p *= .54) had smaller effects and in the opposite direction; it appears that they operated as expected only through their influence on Motivation. The indirect paths mediated by motivation were statistically significant, that is, Opportunity→ Motivation → Behaviour (*b* = .60, *p *< .001); Capability → Motivation → Behaviour (*b* = .10, *p *< .001). As we observed in the OLS regression models, several exogenous socio‐demographic control variables were statistically significant. Notably, age (*b* = .07, *p *< .01) and income (*b* = .13, *p *< .001) were associated with an increase in hygienic practices, whilst being male (*b *= −.18, *p *< .001), non‐Christian (*b *= −.14, *p *< .001), non‐religious (*b *= −0.12, *p *< .001), and suburban (*b *= −.08, *p *< .01) were associated with a decrease in hygienic practices.

## Discussion

This research explored UK citizens’ hygienic practices during the early stage of the COVID‐19 pandemic in relation to the COM‐B model of behaviour change (Michie *et al.*, 2011). We found that all three COM‐B components significantly predicted good hygienic practices, with motivation having the greatest influence on behaviour. Our subscale analysis revealed that automatic motivation and physical opportunity negatively influenced behaviour, whilst psychological capability, social opportunity, and reflective motivation positively influenced behaviour. The reflective motivation subscale was largely driving behaviour. Our SEM represents the nature and strength of the relationships between constructs, providing useful insight into the pathways to behaviour.

Our findings have clear implications for the design of behaviour change interventions (BCIs) to promote hygienic practices. First, interventions should focus on increasing and maintaining motivation to act and contain behaviour change techniques (BCTs) that focus on self‐regulatory processes involving planning and goal setting. We suggest utilizing implementation intentions, a specific planning technique found to help successfully bridge the ‘intention‐behaviour’ gap (Gollwitzer, 1999; Golwitzer & Sheeran, 2006). Further, since individuals will need to have the ability to enact such techniques themselves during the lockdown, we suggest utilizing the compendium of self‐enactment BCTs (Knittle *et al.*, 2020) in intervention design (self‐regulatory techniques #5 ‐ #18 are especially relevant). Second, any intervention might correspondingly include elements that promote and maintain social support and knowledge of COVID‐19 transmission. Third, our data show that groups in particular need of targeting for interventions to increase hygienic practices are males and those living in cities and suburbs, and individuals who do not have religious beliefs. Fourth, we would recommend paying attention to the ways in which COM‐B components impact upon behaviour through their effects upon motivation – it is unlikely, for example, that promoting the opportunity to act will promote the enactment of hygienic practices. Fifth, consideration must be given to promoting the maintenance of these behaviours over time and making the transition from lockdown as social isolation measures are lifted. Finally, consideration of the political and social circumstances would inform intervention designers of the impact of other influences on behaviour – for example, public health campaigns.

This study has both strengths and limitations. This is the first study to our knowledge to explore hygienic practices in relation to the current pandemic in the United Kingdom; we have captured the early behavioural response to the lockdown in a highly representative sample of the UK population and can make concrete recommendations for the design of interventions to help promote hygienic practices. Whilst there is a wide and good‐quality literature on the enactment of hygiene behaviour, especially handwashing (Lunn *et al.*, 2020), this study adds to our understanding of these behaviours in the current context where the drivers of behaviour and nature of the threat may be entirely different from usual circumstances.

However, we are aware that despite the sampling frame and large sample size, it was not a true random probability sample (which would have been very difficult to obtain under the current circumstances) and it is possible that individuals’ decisions about whether to participate were affected by psychological factors, creating the possibility of sampling bias. Also, the usual limitations of self‐report apply in terms of the possibility of reporting bias. In usual circumstances, further in‐depth qualitative work would be conducted addressing barriers and enablers to behaviour and this would feed into intervention design and content (as proposed, for example, by French *et al.*, 2012). We hope that future waves of the survey, mapping changes in behaviour, will help us gain further insight into the particular difficulties facing UK citizens in this context.

Understanding the determinants of individual behaviour is paramount to mitigating the severity and progress of the coronavirus outbreak. This study provides important insights into the influences on people’s hygienic practices during the pandemic and will help to inform strategies for intervention throughout the isolation period and beyond.

## Conflicts of interest

All authors declare no conflict of interest.

## Supporting information


**Appendix S1** **Table S1** **.** Descriptive data for survey items used to measure hygienic practices and COM‐B.

**Appendix S2** **Table S2** **.** Descriptive Statistics for Hygienic Practices, COM‐B, and Socio‐Demographic Controls.

**Appendix S3** **Figure S1** **.** The effects of COM‐B components on hygienic practices, controlling for socio‐demographics.
**Appendix S3** **Table S3** **.** OLS regression estimates of COM‐B components predicting hygienic practices (with and without controls).

**Appendix S4** **Table S4** **.** OLS regression estimates of COM‐B sub‐scale models predicting hygienic practices.

**Appendix S5** **Table S5** **.** Results from the full structural equation model (SEM).

## Data Availability

The data that support the findings of this study will be deposited in the data archive 6 months from the end of the study.
