# Supplementary material for: Capability, opportunity, and motivation to enact hygienic practices in the early stages of the COVID‐19 outbreak in the United Kingdom
Source: Br J Health Psychol. 2020 May 16;25(4):856–64. doi: 10.1111/bjhp.12426 (PMC7276910; doi:10.1111/bjhp.12426)
Supplement: Supplementary file 1 — Appendix S1 Table S1 . Descriptive data for survey items used to measure hygienic practices and COM‐B. [file BJHP-25-856-s004.docx]

Appendix 1

Table A1. Descriptive data for survey items used to measure hygienic practices and COM-B

| *Hygienic Practices (3-point scale)* | | *Mean* |
| --- | --- | --- |
| B1 | “Washed your hands with soap and water more often.” | 2.72 |
| B2 | “Used hand sanitising gel if soap and water were not available.” | 2.38 |
| B3 | “Used disinfectants to wash surfaces in your home more frequently.” | 2.22 |
| B4 | “Covered your nose and mouth with a tissue or sleeve when coughing or sneezing.” | 2.64 |
| B5 | Touching eyes and mouth (5-point scale) | 3.62 |
| *COM-B items (5-point scale)* | |  |
|  | *Psychological Capability* |  |
| C1 | “I knew about why it was important and had a clear idea about how the virus was transmitted.” | 4.21 |
| C2 | “I knew about how and when to do it.” | 4.21 |
| C3 | “I was able to overcome the physical and/or mental barriers that might have stopped me from doing it.” | 3.92 |
|  | *Physical opportunity* |  |
| O1 | “I had the necessary time to do it.” | 4.20 |
| O2 | “It was easy for me to do it.” | 4.21 |
|  | *Social opportunity* |  |
| O3 | “People were doing it around me.” | 3.81 |
| O4 | “I had reminders that prompted me.” | 3.46 |
| O5 | “I had support from others.” | 3.55 |
| O6 | “I felt like doing it was normal and expected.” | 4.15 |
|  | *Reflective motivation* |  |
| M1 | “I intended to do it.” | 4.18 |
| M2 | “I felt that I wanted to do it.” | 4.17 |
| M3 | “I believe that it was a good thing to do.” | 4.31 |
| M4 | “I developed a specific plan for doing it.” | 3.36 |
| M5 | “I developed a habit of it in my everyday routine.” | 3.95 |
|  | *Automatic motivation* |  |
| M6 | “It made me feel anxious.” R | 2.68 |
| M7 | “It made me feel disgusted.” R | 2.14 |
| M8 | “I felt like I could control my emotional reactions so I could do it.” | 3.42 |

*Notes:* Higher scores relate to higher compliance/agreement with that measure. R indicates item is reverse-coded.
