# Supplementary material for: Capability, opportunity, and motivation to enact hygienic practices in the early stages of the COVID‐19 outbreak in the United Kingdom
Source: Br J Health Psychol. 2020 May 16;25(4):856–64. doi: 10.1111/bjhp.12426 (PMC7276910; doi:10.1111/bjhp.12426)
Supplement: Supplementary file 2 — Appendix S2 Table S2 . Descriptive Statistics for Hygienic Practices, COM‐B, and Socio‐Demographic Controls. [file BJHP-25-856-s003.docx]

Appendix 2

Table A2. Descriptive Statistics for Hygienic Practices, COM-B, and Socio-Demographic Controls

| Scale | Mean | SD | Median | Min | Max | Alpha |
| --- | --- | --- | --- | --- | --- | --- |
| *Dependent Variable* |  |  |  |  |  |  |
| Hygienic Practices | 0.74 | 0.26 | 0.75 | 0 | 1 | 0.74 |
| *Independent Variables: COM-B* |  |  |  |  |  |  |
| Capability (Psychological) | 0.78 | 0.18 | 0.75 | 0 | 1 | 0.79 |
| Opportunity (Global) | 0.72 | 0.16 | 0.75 | 0 | 1 | 0.78 |
| Opportunity (Psychological) | 0.80 | 0.19 | 0.75 | 0 | 1 | 0.85 |
| Opportunity (Strategic) | 0.69 | 0.18 | 0.69 | 0 | 1 | 0.71 |
| Motivation (Global) | 0.63 | 0.14 | 0.62 | 0 | 1 | 0.65 |
| Motivation (Reflective) | 0.75 | 0.17 | 0.75 | 0 | 1 | 0.81 |
| Motivation (Strategic) | 0.66 | 0.23 | 0.62 | 0 | 1 | 0.66 |
| Motivation (Automatic) | 0.63 | 0.19 | 0.67 | 0 | 1 | 0.31 |
| *Independent Variables: Socio-demographic Controls* |  |  |  |  |  |  |
| Age (Continuous) | 45.45 | 15.90 | 45.00 | 18 | 83 | -- |
| Male (Reference: Female) | 0.48 | 0.50 | 0 | 0 | 1 | -- |
| Non-White (Ref: White British/Irish or White Other) | 0.09 | 0.28 | 0 | 0 | 1 | -- |
| Low Education (No/some quals; ref: University deg. or higher) | 0.13 | 0.34 | 0 | 0 | 1 | -- |
| Moderate Education (O/A-levels; ref: University deg. or higher) | 0.43 | 0.49 | 0 | 0 | 1 | -- |
| Income (Continuous) | 0.50 | 0.36 | 0.50 | 0 | 1 | -- |
| Non-Christian (Ref: Christian) | 0.12 | 0.32 | 0 | 0 | 1 | -- |
| Non-Religious (Ref: Christian) | 0.38 | 0.49 | 0 | 0 | 1 | -- |
| Non-UK Born (Ref: Born in the UK) | 0.09 | 0.29 | 0 | 0 | 1 | -- |
| Personal Risk (Pre-existing cond. or pregnant; Ref: Not at risk) | 0.38 | 0.49 | 0 | 0 | 1 | -- |
| City (Ref: Town) | 0.25 | 0.43 | 0 | 0 | 1 | -- |
| Suburb (Ref: Town) | 0.28 | 0.45 | 0 | 0 | 1 | -- |
| Rural (Ref: Town) | 0.17 | 0.37 | 0 | 0 | 1 | -- |
| Wales (Ref: England) | 0.03 | 0.17 | 0 | 0 | 1 | -- |
| Scotland (Ref: England) | 0.08 | 0.27 | 0 | 0 | 1 | -- |
| N. Ireland (Ref: England) | 0.02 | 0.15 | 0 | 0 | 1 | -- |
