# Supplementary material for: Capability, opportunity, and motivation to enact hygienic practices in the early stages of the COVID‐19 outbreak in the United Kingdom
Source: Br J Health Psychol. 2020 May 16;25(4):856–64. doi: 10.1111/bjhp.12426 (PMC7276910; doi:10.1111/bjhp.12426)
Supplement: Supplementary file 3 — Appendix S3 Figure S1 . The effects of COM‐B components on hygienic practices, controlling for socio‐demographics. Appendix S3 Table S3 . OLS regression estimates of COM‐B components predicting hygienic practices (with and without controls). [file BJHP-25-856-s002.docx]

Appendix 3

Figure A1. The effects of COM-B components on hygienic practices, controlling for socio-Demographics


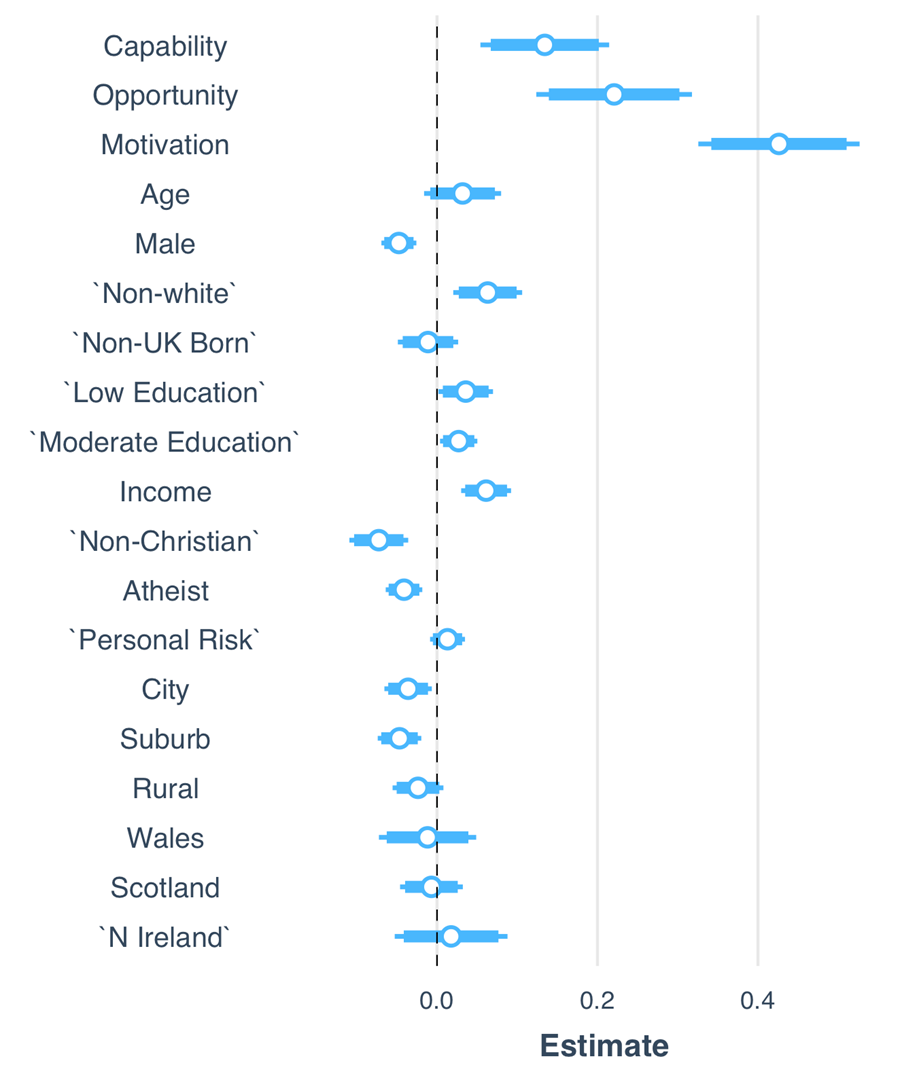


*Notes:* Plot points are unstandardized regression coefficients from an OLS model; 95% and 90% confidence intervals indicated by the narrow and thick error bars, respectively. Plot points to the right of the vertical line indicate an increase in hygienic practices; those to the left a decrease. To aid in interpretation, all predictors have been rescaled from 0 to 1. Using this metric, we see that motivation has the largest effect size relative to the other COM-B sub-scales (b = 0.43; 95% CI = 0.33, 0.53; p < 0.001)

Table A3. OLS regression estimates of COM-B components predicting hygienic practices (with and without controls)

|  | Model 1 | Model 2 |
| --- | --- | --- |
| (Intercept) | 0.18 *** | 0.19 *** |
|  | [0.13, 0.24] | [0.12, 0.26] |
| Capability | 0.14 *** | 0.13 ** |
|  | [0.06, 0.21] | [0.05, 0.21] |
| Opportunity | 0.26 *** | 0.22 *** |
|  | [0.17, 0.36] | [0.12, 0.32] |
| Motivation | 0.42 *** | 0.43 *** |
|  | [0.32, 0.52] | [0.33, 0.53] |
| Age |  | 0.00 |
|  |  | [-0.00, 0.00] |
| Male |  | -0.05 *** |
|  |  | [-0.07, -0.03] |
| Non-white |  | 0.06 ** |
|  |  | [0.02, 0.11] |
| Non-UK Born |  | -0.01 |
|  |  | [-0.05, 0.03] |
| Low Education |  | 0.04 * |
|  |  | [0.00, 0.07] |
| Moderate Education |  | 0.03 * |
|  |  | [0.00, 0.05] |
| Income |  | 0.06 *** |
|  |  | [0.03, 0.09] |
| Non-Christian |  | -0.07 *** |
|  |  | [-0.11, -0.04] |
| Atheist |  | -0.04 *** |
|  |  | [-0.06, -0.02] |
| Personal Risk |  | 0.01  [-0.01, 0.03] |
| City |  | -0.04 * |
|  |  | [-0.07, -0.01] |
| Suburb |  | -0.05 *** |
|  |  | [-0.07, -0.02] |
| Rural |  | -0.02 |
|  |  | [-0.06, 0.01] |
| Wales |  | -0.01 |
|  |  | [-0.07, 0.05] |
| Scotland |  | -0.01 |
|  |  | [-0.05, 0.03] |
| N. Ireland |  | 0.02 |
|  |  | [-0.05, 0.09] |
| *N* | 2025 | 1951 |
| *R^2^* | 0.17 | 0.20 |
| *Notes*: Cell entries contain unstandardized coefficients from OLS regression. 95% Confidence intervals in brackets; **** p < 0.001; ** p < 0.01; * p < 0.05.* | | |
