# Supplementary material for: Capability, opportunity, and motivation to enact hygienic practices in the early stages of the COVID‐19 outbreak in the United Kingdom
Source: Br J Health Psychol. 2020 May 16;25(4):856–64. doi: 10.1111/bjhp.12426 (PMC7276910; doi:10.1111/bjhp.12426)
Supplement: Supplementary file 4 — Appendix S4 Table S4 . OLS regression estimates of COM‐B sub‐scale models predicting hygienic practices. [file BJHP-25-856-s005.docx]

Appendix 4

Table A4. OLS regression estimates of COM-B sub-scale models predicting hygienic practices

|  | Model 3 |
| --- | --- |
| (Intercept) | 0.26 *** |
|  | [0.20, 0.31] |
| Capability (Psychological) | 0.11 * |
|  | [0.02, 0.20] |
| Opportunity (Physical) | -0.08 |
|  | [-0.17, 0.01] |
| Opportunity (Social) | 0.18 *** |
|  | [0.11, 0.25] |
| Motivation (Reflective) | 0.51 *** |
|  | [0.42, 0.61] |
| Motivation (Automatic) | -0.06 * |
|  | [-0.12, -0.00] |
| *N* | 2025 |
| *R^2^* | 0.19 |
| *Notes*: Cell entries contain unstandardized coefficients from OLS regression. 95% Confidence intervals in brackets; **** p < 0.001; ** p < 0.01; * p < 0.05.* | |
