# Supplementary material for: Capability, opportunity, and motivation to enact hygienic practices in the early stages of the COVID‐19 outbreak in the United Kingdom
Source: Br J Health Psychol. 2020 May 16;25(4):856–64. doi: 10.1111/bjhp.12426 (PMC7276910; doi:10.1111/bjhp.12426)
Supplement: Supplementary file 5 — Appendix S5 Table S5 . Results from the full structural equation model (SEM). [file BJHP-25-856-s001.docx]

Appendix 5

Table S5. Results from the full structural equation model (SEM)

Estimator DWLS

Optimization method NLMINB

Number of free parameters 108

Number of observations 2025

Latent Variables:

Estimate Std.Err z-value P(>|z|) Std.lv Std.all

Motivation =~

Q94_10 1.000 0.896 0.896

Q94_11 0.990 0.007 136.318 0.000 0.887 0.887

Q94_12 0.991 0.008 124.075 0.000 0.888 0.888

Q94_13 0.549 0.016 33.733 0.000 0.492 0.492

Q94_14 0.827 0.011 72.466 0.000 0.741 0.741

Q94_17 0.449 0.019 23.827 0.000 0.402 0.402

Capability =~

Q94_1 1.000 0.882 0.882

Q94_2 1.023 0.010 101.680 0.000 0.902 0.902

Q94_3 0.812 0.014 57.368 0.000 0.716 0.716

Opportunity =~

Q94_4 1.000 0.863 0.863

Q94_5 1.008 0.010 103.549 0.000 0.870 0.870

Q94_6 0.762 0.015 51.807 0.000 0.657 0.657

Q94_7 0.545 0.017 31.530 0.000 0.470 0.470

Q94_8 0.645 0.016 39.178 0.000 0.557 0.557

Q94_9 0.993 0.010 95.504 0.000 0.857 0.857

Behaviour =~

Q87_2 1.000 0.938 0.908

Q87_3 0.845 0.031 26.966 0.000 0.793 0.774

Q87_4 0.775 0.031 25.400 0.000 0.727 0.713

Q87_5 0.844 0.034 24.920 0.000 0.792 0.774

Q86_8 0.297 0.032 9.289 0.000 0.279 0.278

Regressions:

Estimate Std.Err z-value P(>|z|) Std.lv Std.all

Motivation ~

Capability (a) 0.131 0.028 4.755 0.000 0.129 0.129

Opportunity (b) 0.852 0.027 31.388 0.000 0.820 0.820

Behaviour ~

Capability -0.042 0.069 -0.612 0.541 -0.039 -0.039

Opportunity -0.260 0.130 -2.000 0.046 -0.239 -0.239

Motivation (c) 0.771 0.115 6.732 0.000 0.737 0.737

Age 0.004 0.002 2.592 0.010 0.005 0.073

Male -0.338 0.051 -6.622 0.000 -0.360 -0.180

Non-White 0.173 0.096 1.803 0.071 0.184 0.052

Non-Christian -0.401 0.083 -4.820 0.000 -0.427 -0.137

Atheist -0.224 0.053 -4.233 0.000 -0.238 -0.116

Income 0.085 0.018 4.760 0.000 0.090 0.129

Low Education 0.127 0.076 1.669 0.095 0.135 0.046

Mod Education 0.082 0.055 1.507 0.132 0.088 0.043

City -0.080 0.064 -1.264 0.206 -0.086 -0.037

Suburb -0.159 0.057 -2.799 0.005 -0.170 -0.076

Covariances:

Estimate Std.Err z-value P(>|z|) Std.lv Std.all

Capability ~~

Opportunity 0.659 0.011 58.991 0.000 0.866 0.866

Indirect Effects:

Estimate Std.Err z-value P(>|z|) Std.lv Std.all

a*c 0.101 0.026 3.892 0.000 0.095 0.095

b*c 0.657 0.100 6.568 0.000 0.604 0.604
